# NQS-Doped PDMS Solid Sensor: From Water Matrix to Urine Enzymatic Application

**DOI:** 10.3390/bios11060186

**Published:** 2021-06-08

**Authors:** Adrià Martínez-Aviñó, Lusine Hakobyan, Ana Ballester-Caudet, Yolanda Moliner-Martínez, Carmen Molins-Legua, Pilar Campíns-Falcó

**Affiliations:** MINTOTA Research Group, Department of Analytical Chemistry, University of Valencia, Dr. Moliner 50, Burjassot, 46100 Valencia, Spain; adria.martinez@uv.es (A.M.-A.); lusine.hakobyan@uv.es (L.H.); yolanda.moliner@uv.es (Y.M.-M.); carmen.molins@uv.es (C.M.-L.); pilar.campins@uv.es (P.C.-F.)

**Keywords:** ammonium, urea, urease, urea hydrolysis, NQS-PDMS sensor, optical sensor, glass support, in-situ analysis, water, urine

## Abstract

The development of in situ analytical devices has gained outstanding scientific interest. A solid sensing membrane composed of 1,2-naphthoquinone-4-sulfonate (NQS) derivatizing reagent embedded into a polymeric polydimethylsiloxane (PDMS) composite was proposed for in situ ammonium (NH_4_^+^) and urea (NH_2_CONH_2_) analysis in water and urine samples, respectively. Satisfactory strategies were also applied for urease-catalyzed hydrolysis of urea, either in solution or glass-supported urease immobilization. Using diffuse reflectance measurements combined with digital image processing of color intensity (RGB coordinates), qualitative and quantitative analyte detection was assessed after the colorimetric reaction took place inside the sensing membrane. A suitable linear relationship was found between the sensor response and analyte concentration, and the results were validated by a thymol-PDMS-based sensor based on the Berthelot reaction. The suggested sensing device offers advantages such as rapidity, versatility, portability, and employment of non-toxic reagents that facilitate in situ analysis in an energy-efficient manner.

## 1. Introduction

The development of in-situ analytical devices to estimate ammonium concentration in environmental samples is of scientific interest due to the environmental problems associated with intensive agricultural and animal farming activities [1,2,3]. The growing concern for climate change has focused its attention on emissions and discharges of NH_3_/NH_4_^+^ [4,5] as the prevention of their negative effects has led to legal requirements at regional, national, and European levels [6] as well as the need for working conditions to which these industries must adapt [7]. Hence, the development of methods for real-time and in situ measurements has become mandatory to facilitate the decision-making process [8].

The in situ analysis devices that have become the most trending analyzers are based on colorimetric tests employing chemical sensors as support [9]. For instance, colorimetric sensors supported on polymeric bases, such as polydimethylsiloxane (PDMS) [10,11], paper [12], and nylon [13], are the most commonly used sensing devices that improve the sustainability aspects of the analytical method. Among them, the PDMS matrix has already proved to be a satisfactory silicon-based organic base for colorimetric purposes [14]. This is the case of an NQS-doped PDMS-based sensor in which 1,2-naphthoquinone-4-sulfonate (NQS) is entrapped in PDMS using a sol–gel process [15]. The combination of the hydrophobicity provided by PDMS with the hydrophilicity of tetraethyl orthosilicate (TEOS) in the presence of SiO_2_ NPs gives rise to a promising nanomaterial with extra functionality. In fact, previous research has demonstrated that when TEOS and SiO_2_ NPs are incorporated in NQS-doped PDMS sensors and exposed to amino compounds in gaseous samples, the amino compounds diffuse into the hybrid polymeric matrix, and a color change takes place owing to the formation of the NQS derivative inside the membrane [16,17]. This sensing performance led to a satisfactory in-situ analysis device for the determination of the primary and secondary amine biocide *N*-(3-aminopropyl)-*N*-dodecyl-1,3-propanediamine in industry [18].

New strategies were investigated to improve the sensitivity of the NQS-PDMS-based sensor. In fact, sensing properties were tuned in the presence of ionic liquids (ILs) such as 1-methyl-3-octylimidazolium hexafluorophosphate, 1-butyl-4-methylpyridinium hexafluorophosphate, and 1-butyl-3-methylimidazolium octyl sulfate. These organic cations and anions show unique structural and physiochemical characteristics that yield to specific interactions inside the membrane [19,20,21]. Specifically, ILs endowed an improvement on the mechanical properties of the PDMS-TEOS-SiO_2_ NP composite, maintaining flexibility and tractability [22,23] and improving composite porosity. Among the potential applications of the PDMS/TEOS-SiO_2_ NP–IL membrane, its optimal analytical features for detecting gaseous ammonia and amines during meat spoilage are worth mentioning [24]. The compatibility of the NQS-based PDMS/TEOS/SiO_2_ NPs composite in an aqueous solution was assessed and confirmed to provide satisfactory analysis, for instance, in the control of excess casein in effluents from dairy industries [14]. The PDMS/TEOS-based sensing membrane was proposed as a promising alternative for in situ analysis in solutions thanks to the portability and the minimization of reagents handling; therefore, it is worth exploiting its full potentiality.

Urea is a nitrogen-containing compound that is also present in the environment, coming from agricultural processes (soil) [25,26] and water [27]. Typically, urea is employed as fertilizer that can be indirectly determined as ammonia after urea hydrolysis reaction. In addition to environmental pollution, urea detection is relevant in medical diagnosis, since urea is one of the main waste products of protein and amino acid metabolism and is eliminated from the body through urine [28]. Urine ammonium compounds excreted by the human body can be considered as remarkable biomarkers for indicating diseases or pathophysiological conditions [29,30,31]. At a physiological pH (7.3), urea is neutral, with an expected concentration in the range of 155–388 mM (9.3–23.3 g/L) in humans [32]. In the literature, several analytical methods for the determination of urea use enzymatic hydrolysis, which is carried out in the presence of the urease enzyme in the working medium [33,34,35,36]. Usually, enzymatic assays are performed in a solution, and there are several immobilization strategies [37] on different supports [31,33,35,38]. The immobilization process is a key factor to develop efficient sensing devices with good operational and storage stability, high selectivity, short response time, and high reproducibility. Particularly, a glass surface has been used as support for biofunctionalization with aminopropyltrimethoxysilane (APTMS) [39,40]. The terminal group of APTMS provides a reactive residue to bind biomolecules or crosslinkers to the support surface, giving rise to successful covalent immobilization of urease. A comparative study on urea hydrolysis when urease is in solution or immobilized on a solid support is of interest, with the aim of determining urease stability and reusability. Among the samples applied, selective urea nitrate in solution was performed in a polymer matrix composed of poly(acrylamide) hydrogel [41], and the employment of portable, low-cost devices for in situ determination of urea has already been addressed in milk [42] and blood [43] matrices.

In this work, we further explore the use of the NQS-based PDMS/TEOS/SiO_2_ NP composite as an ammonia sensor to detect NH_4_^+^ and urea in water and human urine samples, respectively. Moreover, urea enzymatic hydrolysis containing urease in solution and immobilized on glass support were investigated. The NQS-based colorimetric sensing device proposed in this work offers a sustainable alternative that possesses advantages such as versatility, simplicity, rapidity, satisfactory robustness over the reagent derivatizations in solution, cost-effectiveness, energy-efficiency, and reliable response that can be detected visually.

## 2. Materials and Methods

### 2.1. Materials and Reagents

PDMS membranes were synthesized using a Sylgard^®^ 184 Silicone Elastomer Kit (base and curing agent) obtained from Dow Corning (USA). Sodium 1,2-naphthoquinone-4-sulfonate (NQS, 99.7%), tetraethyl orthosilicate (TEOS ≥ 99.0%), silicon dioxide nanoparticles (SiO_2_ NPs, 99.5%, 5–15 nm particle size), urease (*Canavalia ensiformis*–*Jack bean* 64,347 units/g in 0.31 g), and APTMS (aminopropyltrimethoxysilane) were purchased from Sigma Aldrich (St. Louis, MO, USA). Sodium carbonate, sodium hydrogen carbonate, and ammonium chloride were provided by Probus (Spain). Urea, hydrogen peroxide, 2-propanol (isopropanol ≥ 99.9%), and sodium hydroxide were provided by VWR Chemicals (Radnor, PA, USA). Trichloroacetic acid (≥99.0%) and sodium dihydrogen phosphate monohydrate were obtained from Merck (Darmstadt, Germany). Hydrochloric acid 37% was obtained from Scharlau (Barcelona, Spain). Di-sodium hydrogen phosphate anhydrous was obtained from Panreac (Barcelona, Spain). Ultrapure water was obtained from a Nanopure II system (Sybron, MS, USA). Solid-glass beads (borosilicate glass balls, diam. 5 mm) were provided by Sigma Aldrich (St. Louis, MO, USA). A stock urease solution of 9 mg mL^−1^ was prepared in phosphate buffer (pH 7.4) and stored in darkness at 4 °C. Phosphate buffer was prepared from 0.2 M NaH_2_PO_4_ and Na_2_HPO_4_, and the pH was adjusted to 7.4 with sodium hydroxide 1 M. Carbonate buffer was prepared from 0.1 M NaHCO_3_ and Na_2_CO_3_, and the pH was adjusted to 11 with sodium hydroxide 1 M.

Thymol was purchased from Riedel-de Haen (Munich, Germany). Thymol solution was prepared by dissolving an adequate amount of the solid reagent in acetone. Sodium hypochlorite and nitroprusside were obtained from Probus (Badalona, Spain).

### 2.2. Apparatus

Solid sensor morphology was studied with a Hitachi S-4800 scanning electron microscope at an accelerating voltage of 20 keV over metalized samples with a mixture of gold and palladium for 30 s.

A Nikon ECLIPSE E200LED MV Series optical microscope (Nikon Corporation, Tokyo, Japan) was employed under bright-field illumination using a 50× objective to characterize the PDMS membrane. Nis Elements 4.20.02 software (Nikon Corporation) was used for acquiring the images.

Absorbance measurements were carried out using a Cary 60 UV–vis spectrophotometer equipped with a diffuse reflection probe from Harrick Scientific Products (Pleasantville, NY, USA). The diffuse reflection probe has an integral video camera, which provides a visual image to select the sample spot to be analyzed. Spectra were recorded from 400 to 900 nm. For data collection and processing, Cary WinUV Scan application 5.0.0.999 software from Agilent Technologies was used.

For UV–vis measurements in the solution, an HP-8453 UV–vis spectrophotometer from Hewlett Packard (Poway, CA, USA) furnished with a 1-cm path length quartz microcell was employed. Absorption spectra were registered from 190 to 900 nm.

A smartphone coupled to a miniaturized spectrometer (GoSpectro, ALPHANOV, Talence, France) was employed for registering the absorbance spectrum in the working range from 400 to 900 nm. Sensor digital images were taken using a smartphone, and red, green, and blue coordinates (RGB) were obtained.

### 2.3. Preparation of PDMS/TEOS-NQS-SiO_2_ NPs Sensing Membranes

**Figure 1:**
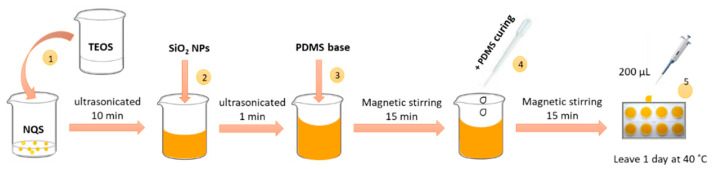
Steps of the sol-gel process that takes place for the sensor preparation.

The fabrication of the PDMS/TEOS-SiO_2_ NPs composite with NQS reagent [17] was carried out following the experimental procedure described in Figure 1. First, the derivatizing NQS (0.35%) was mixed with TEOS (59%) and ultrasonicated for 10 min. Then, SiO_2_ NPs (0.41%) were added to the NQS-TEOS suspension, and the mixture was ultrasonicated for 1 min to completely dissolve the NPs. The PDMS elastomer base (36.5%) was added to the resulting NQS-TEOS-SiO_2_ NP mixture under vigorous stirring for 15 min. Drops of PDMS curing agent (3.65%) were then added. Finally, 200 µL of the homogeneous mixture was deposited on plastic well-plates (d = 1.5 cm) for gelation. The gelation procedure took place at 40 °C for 24 h.

### 2.4. Analytical Response Measurements

#### 2.4.1. Ammonium Measurement in Aqueous Matrix

The measurement of NH_4_^+^ in aqueous matrices was performed by introducing the sensing membrane into a vial containing 1 mL of NH_4_^+^ solution (NH_4_^+^ standards, water samples were diluted if necessary) and 1 mL of carbonate buffer (pH 11). The solution was then heated at 100 °C for 10 min. Finally, the sensing device was removed from the solution and the color change was measured by diffuse reflectance response. The color change of the sensor was easily observed by visual inspection.

#### 2.4.2. Urea Measurement in Urine Matrix

Initially, protein precipitation of urine samples obtained from healthy volunteers was performed by adding 5 mL of 15% trichloroacetic acid to a 10 mL urine sample, and the mixture was left to stand for 5 min. The mixture was then centrifuged for 10 min at 3500 rpm and the precipitate-free liquid was taken and stored at 4 °C under dark conditions until the moment of the analysis. A quantity of 100 µL of urea standards or urine samples was introduced into a vial containing 1 mL of carbonate buffer (pH 11). A fourth part of the sensor was placed inside the vial and the mixture was heated at 100 °C for 10 min. Finally, the sensor was removed from the solution and its response was quantitatively measured by diffuse reflectance and semi-qualitatively measured by visual inspection.

#### 2.4.3. Direct Urea Hydrolysis

In order to hydrolyze urea from urine samples, a 1:10 dilution step with deionized water was performed. Urease solution was prepared by dissolving 9 mg of the enzyme in 1 mL phosphate buffer (pH 7) and stored at 4 °C in the dark until use. Then, 100 µL of the diluted urine solution was mixed with 1 mL of phosphate buffer (pH 7.4) in an Eppendorf tube, and 20 µL of urease solution was added. The resulting solution was heated at 37 °C for 5 min in a water bath. The analytical assay was performed as described in Section 2.4.2 using the proposed sensor and the hydrolyzed urine solution (or NH_4_^+^ standard).

#### 2.4.4. Solid Supported Urea Hydrolysis

The functionalization mechanism for covalent urease enzyme immobilization on borosilicate glass balls support (5 mm diameter) was carried out following a combination of experimental procedures found in the literature for silicate-based materials [44,45,46]. Initially, glass balls were treated with a mixture of H_2_O:HCl (37%):H_2_O_2_ (30%) (5:1:1) and continuously stirred for 2 h. Afterward, the mixture was removed, and the glass balls were washed with deionized water and air-dried. On the other hand, a solution composed of 1 mL of aminopropyltrimethoxysilane (APTMS), 5 mL of 0.1 M acetic acid, and 5 mL of isopropanol was prepared in a plastic beaker and stirred for 2 h. Then, 89 mL of isopropanol was added to the mixture, which was poured on the glass balls and left at 60 °C until dry. Finally, the glass balls were immersed in 300 μL of urease solution (see preparation in Section 2.4.3) and the mixture was stirred for 1 h. The remaining solution was eliminated, and the glass balls were ready to be used.

The urea hydrolysis procedure consisted of mixing 100 µL of the diluted urine solution with 1 mL of phosphate buffer (pH 7.4). Then, urease immobilized on the glass balls was added to the solution, and the mixture was heated at 37 °C for 5 min in a water bath. Glass supports were removed from the solution, and the hydrolyzed urine (or NH_4_^+^ standard) was analyzed following the experimental procedure described in Section 2.4.2.

## 3. Results

### 3.1. Performance of Solid Supported NQS-PDMS/TEOS-SiO_2_ NPs for Ammonium and Urea

**Figure 2:**
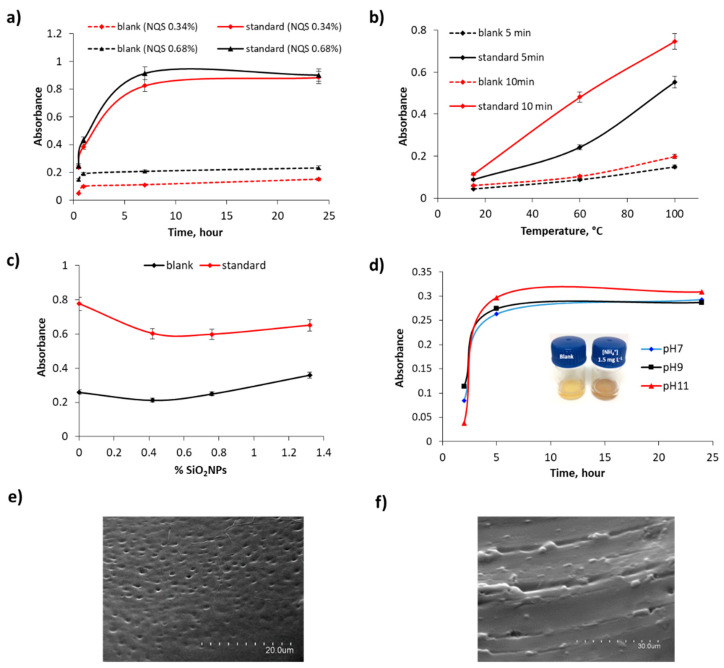
NH_4_^+^ (50 mg L^−1^) standard solutions: (**a**) Variation of the colorimetric signal as a function of the NQS content and exposure time at room temperature. (**b**) Variation of the analytical signal as a function of the temperature for 5 and 10 min reaction times. (**c**) Variation of sensor response depending on the SiO_2_ NP composition tested. (**d**) Analytical response as a function of the pH and exposure time; inset: images of the sensing device in blank solution (yellow) and NH_4_^+^ standard solution 1.5 mg L^−1^ (brownish) after 5 h exposure time at room temperature. (**e**,**f**) SEM images of the synthesized NQS-PDMS sensing membrane, scale bar: 20 µm (**e**) and 30 µm (**f**).

The ammonium content, detected as ammonia by the chemical sensor, and urea were determined by the proposed sensing device. Colorimetric detection occurs when the target analyte diffuses along the porous PDMS/TEOS membrane and reacts with the colorimetric probe, NQS, embedded inside the sensing membrane, yielding a colorimetric signal that changes from orange to a brownish color [16]. For quantification purposes, initial studies were carried out in aqueous standards (NH_4_^+^ = 50 mg L^−1^) using the sensing membrane at room temperature and basic working conditions (pH 7) during sufficient reaction time to allow the colorimetric signal to evolve. Figure 2a shows the variation of the analytical response as a function of the content of the colorimetric dye. Experimentally, it was observed that a colorimetric dye content higher than 0.34% favored the diffusion of the derivatizing analyte toward the solution. In addition to this, no significant enhancement of the sensitivity was observed for the highest dye content during a 24 h reaction time, as can be seen in Figure 2a. Thus, 0.34% NQS content was selected as the optimal composition for the sensor.

On the other hand, it was found that more than 5 h were required for the colorimetric signal to reach an adequate sensitivity level. In an attempt to reduce the reaction time and improve the sensitivity, a thermal treatment was evaluated, since changes in the reaction temperature can change the adsorption behavior and thereby affect the sensitivity. To this end, three different temperatures (15, 60, and 100 °C) at two reaction times (5 and 10 min) were studied, as shown in Figure 2b. The results suggested that an increased temperature drastically reduced the reaction time to achieve a given sensitivity, and therefore would be advantageous for practical application. However, the thermal treatment also induced a loss of physical robustness of the sensing membrane. With the aim of improving the thermal stability of the membrane, SiO_2_ NPs were tested as dopants [47]. The results indicated in Figure 2c confirmed that the presence of SiO_2_ NPs enhanced the stability of the sensors in the thermal treatment, as the sensing membrane deformation was completely avoided at a percentage of 0.4%. Moreover, the presence of SiO_2_ NPs also helped prevent the leaching of the colorimetric dye to the solution at high temperatures, most likely because the NPs acted as additional adsorption sites. Additionally, the sensing device performance was examined under different pH conditions (pH 7, 9, and 11) in order to determine the extent to which the membranes respond to these changes and find the optimal working conditions. As depicted in Figure 2d, the sensor response increased as a function of pH, as the NQS reaction is favored at higher pH [18]. Therefore, pH 11 was chosen as the best working condition. Scanning electron microscopy (SEM) was employed for the characterization of the proposed sensing membrane. Taking into account the optimal synthetic as well as performance conditions of the PDMS sensor, SEM images of the reference sensor (blank) were obtained. The morphology of the PDMS polymeric matrix exhibited noticeable porosity, as shown in Figure 2e. In addition, the presence of SiO_2_ NPs confers the stability of the sensor in the thermal treatment, enhancing its mechanical properties (see Figure 2f).

Interday and intraday precision of the proposed sensor were evaluated by the relative standard deviation (%RSD) values. In this context, the same batch precision study was performed, and the analytical response of the sensor was evaluated. Satisfactory interday (7.25%) and intraday (7.83%) RSD values were obtained. Regarding batch-to-batch precision, intraday RSD values were lower than 8%, and the interday RSD value was 6%, which are satisfactory precision RSD values. Additionally, it should be noted that the sensing membrane was stable as the sol-gel methodology guarantees the stability of the guest molecules as well as the reaction products. The stiffness of the silica matrix prevented the polymeric matrix of agglomerations and guest molecule leaching. These results bear evidence to the fact that the proposed sensing membrane is a reliable and reproducible device to achieve an estimation of NH_3_/NH_4_^+^ in an aqueous medium.

### 3.2. Sensitivity for Ammonium and Urea Standard Solutions

**Figure 3:**
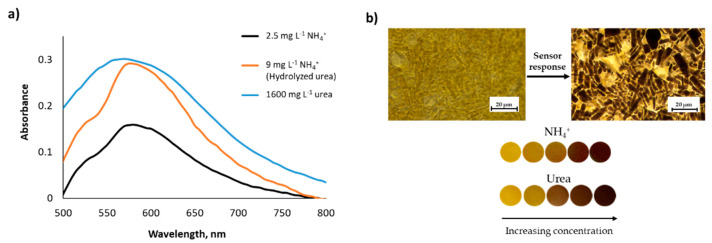
(**a**) Difference in UV-Vis spectra between standards of 2.5 mg L^−1^ NH_4_^+^ (black line), 9 mg L^−1^ NH_4_^+^ coming from hydrolyzed urea (orange line), and 1600 mg L^−1^ urea (blue line) and the blank standard. (**b**) Top: Optical microscopy images of the PDMS membrane before (left) and after (right) being exposed to ammonium/urea in solution, scale bar: 20 µm. Bottom: Sensor photos referring to the evolution of the sensor color as a function of ammonium or urea concentration in solution.

The performance of the NQS colorimetric probe encapsulated within the PDMS matrix was investigated in the presence of NH_4_^+^ (expressed as NH_3_) and amine groups of urea standards, CO(NH_2_)_2_ in an aqueous solution. NH_4_^+^ and urea standards were prepared following the experimental procedures described in Section 2.4.1 and Section 2.4.2, respectively. Reflectance diffuse measurements when using a conventional spectrophotometer were registered, and absorption spectra profiles were obtained. A maximum absorption was found at 590 nm for ammonium and urea standards when representing the difference in UV–visible spectra with respect to the blank standard, as shown in Figure 3. Calibration curves obtained for both analytes, listed in Table 1, indicated satisfactory sensitivity and good linearity of the proposed NQS-based sensor. These results suggest that the NQS-doped PDMS sensor can be a potential candidate for monitoring ammonium and urea in water and urine samples, respectively.

### 3.3. Sensor Device Performance for Hydrolyzed Urea: Urease in Solution vs. Immobilized on Borosilicate Glass Balls

We further explored the applicability of the sensing device for monitoring ammonia generated in situ in the working medium when enzymatic catalysis of urea takes place in the presence of urease [36]. This enzyme hydrolyzes urea into ammonium, and the catalytic reaction typically occurs in solution. Urease solution was prepared as described in Section 2.4.3. The optimal reaction conditions employed were 37 °C and a 5 min reaction time. Regarding urease solution, several urease volumes were assayed in order to determine the optimal urease quantity for hydrolyzing 5.3 mg L^−1^ of urea. As listed in Table 2, a 20 µL volume was found to be the most appropriate to perform the analysis as the sensor response was not significantly enhanced for higher urease volumes. In these conditions, the urease enzyme was capable of hydrolyzing an amount of urea equivalent to 6.2 mg L^−1^ of ammonia in solution. Satisfactory accuracy values near 100% were obtained.

**Figure 4:**
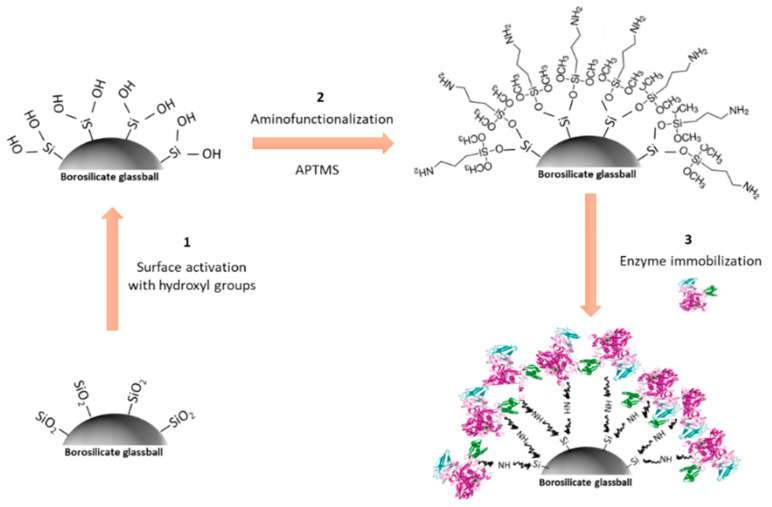
Schematic diagram of covalent urease immobilization on a borosilicate glass surface involving surface functionalization with APTMS.

By fixing the previous reaction conditions, urea hydrolysis was studied following the experimental procedure from Section 2.4.3. The calibration curve obtained for hydrolyzed urea in solution was obtained in a conventional spectrophotometer and is indicated in Table 1. A similar slope was found for both NH_4_^+^ and hydrolyzed urea determinations, which broadens the applicability of the ammonia sensor for detecting both ammonium and hydrolyzed urea. Moreover, the catalytic reaction was investigated in the presence of urease enzyme when it was immobilized on a glass support [37]. Borosilicate glass balls were selected as a solid support on which urease covalently bonded by following the experimental procedure described in Section 2.4.4. Mainly, the strategy for urease immobilization was based on three steps, depicted in Figure 4: (1) glass balls were treated with H_2_O:HCl:H_2_O_2_ (5:1:1) to eliminate contaminants and activate the glass surface with hydroxyl groups [16,46,48]; (2) the activated glass surface was aminofunctionalized with a mixture of APTMS, acetic acid, and isopropanol; and (3) enzyme immobilization covalently bonded to the APTMS by means of the –NH_2_ groups.

**Figure 5:**
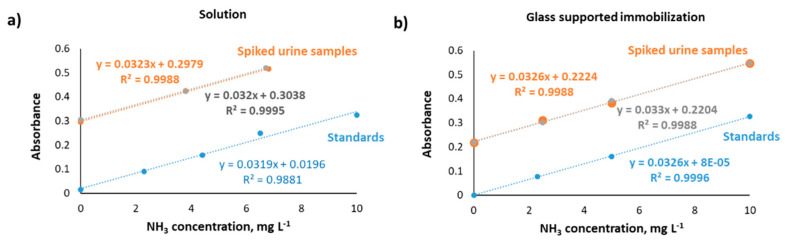
Calibration curves for hydrolyzed urea standards (blue) and spiked urine samples (*n* = 2, orange and grey) for urea catalysis by means of (**a**) urease enzyme in solution and (**b**) glass-supported urease immobilization.

The sensor performance was investigated in the presence of either urease in solution or immobilized on a glass surface. Diffuse reflectance measurements were performed for hydrolyzed urea standards, and calibration slopes were compared. As shown in Figure 5, the calibration slope of urease in solution was 0.0333 ± 0.0015 L mg^−1^, and 0.033 ± 0.0004 L mg^−1^ was obtained for immobilized urease. This result implies that urea hydrolysis is equally effective for both types of experiments. Hence, it is worth mentioning that the immobilization process accounts for an interesting alternative when urease stability and reusability are considered. Additionally, spiked urine samples (*n* = 2) were analyzed, and similar slopes were obtained for the samples and both urea hydrolysis strategies, as plotted in Figure 5. This implies that no matrix effects were present for hydrolyzed urea determination, making the proposed sensing device a potential candidate for in situ detection.

## 4. Proof of Concepts

### 4.1. Water Matrix

Ammonium was monitored in water samples coming from two different water treatment plants (region of Valencia). Samples were analyzed as described in Section 2.4.1. In the case of water treatment plant 1, the found concentrations and colorimetric response for the entrance, decantation, and exit stages are given in Table 3. The colorimetric sensor response and absorbance signals were in agreement with the expected results, that is, the lowest ammonium concentration was found at the exit stage of the treatment plant. In addition to these water samples, wastewaters subjected to oxic and anoxic treatment processes were also studied with the proposed sensing device. The found concentrations calculated for samples S1 and S2 (anoxic treatment reactor input and output, respectively) and samples S3 and S4 (oxic treatment reactor input and output, respectively) were in agreement with the treatments undergone by the samples in the different reactors (see Table 3). In fact, the main difference between the oxic and anoxic processes is the employment of oxygen during water treatment. Typically, anoxic treatment processes are used for the treatment of waste that has a high concentration of biodegradable organic material. Additionally, a recovery study was carried out by analyzing spiked samples with NH_4_^+^ 1.5 mg L^−1^. As listed in Table 3, these values were also satisfactory, involving no significant matrix effects in ammonium determination.

### 4.2. Urine Matrix: Direct Urea and Urease-Catalyzed Hydrolysis Sensing

Direct urea analysis was assayed considering the possible interferences of the nitrogen-based contribution provided by proteins in urine. For the sake of comparison, different urine samples with and without protein precipitation pre-treatment were analyzed. As shown in Table 4, absorbance values for untreated urine were 0.6615 and 0.5654, whereas values of 0.3869 and 0.2895 were obtained for deproteinized urine samples. These results suggest that the presence of proteins in urine samples interfered with direct urea detection, giving rise to an overestimation of the nitrogen-based content. Hence, protein precipitation was considered an essential pre-treatment step for eliminating protein interference following the experimental procedure described in Section 2.4.2. The sensor response was analyzed, and urea concentration was quantitatively determined, as listed in Table 4. The results obtained fall in the upper part of the expected human urea range [32].

Sensor performance was also assessed when urease-catalyzed hydrolysis of urea took place in urine samples. In this case, the sample pre-treatment step consisted of diluting the urine without the protein precipitation procedure. In fact, the blank sensor response (0.2273) was similar to sample responses (0.2169 and 0.1974), which implied the absence of protein interference. Therefore, different pretreatment steps are required depending on the target analyte, that is, urea or hydrolyzed urea. The quantitative analysis yielded the urea concentrations indicated in Table 5. Similar urea concentrations were found for both types of enzymatic catalysis, that is, urease in solution or urease immobilized on a glass support. These results imply that the proposed sensor is reliable and independent of the conditions of the catalytic process, giving rise to a robust in situ analytical method.

## 5. Validation of NQS-Based Sensing Device: Thymol Sensor

With the aim of proving NQS-based sensor reliability, a validation study of the accuracy of urea content in urine samples was carried out using a PDMS composite containing the reagents of the Berthelot reaction, which are released into the reaction medium [10]. Due to the fact that this colorimetric reaction occurs in solution, urea hydrolysis was only carried out in the presence of urease enzyme immobilized on the spherical glass support. Following the measurement procedure described in reference [10], the typical indothymol blue band at 690 nm appears after 5 min reaction time and can be visually detected due to the color change from yellow to green. The absorbance of the solution (hydrolyzed urea standard or urine sample) was registered, and a calibration curve was obtained. Here, hydrolyzed fortified samples were also represented. Similar calibration slopes were found for both types of samples, as listed in Table 6. As stated for the proposed NQS-based sensor, the absence of a matrix effect in urea determination was demonstrated by the thymol sensor. The estimated urea concentration was 10.0 ± 2 g L^−1^ for the same urine sample from Table 5. As can be seen, this result is similar to the mean value (12.5 ± 0.3 g L^−1^) obtained with the NQS sensor, which validates the reliability of the NQS-based sensing device.

## 6. In Situ Analysis

Taking advantage of the portability provided by the NQS-based sensor, we further explored the feasibility of performing quantitative in situ analysis using portable instrumentation [8]. Lately, smartphones have become the most popular intelligent device that offers advantages such as portability and a user-friendly operating system [49,50]. In this context, a smartphone coupled to a miniaturized spectrometer was selected as a portable measuring device for detecting ammonium in wastewater samples. The calibration equation parameters were obtained and compared to those obtained by a conventional spectrophotometer. The results are listed in Table 1. Values of 0.035 and 0.0316 L mg^−1^ for the slope were obtained for conventional and portable spectrometers, respectively. This result suggests that it is possible to perform reliable absorbance measurements using portable instrumentation, which improves the effectiveness of in-situ analysis. Moreover, digital image analysis was also exploited. The calibration curve was obtained by registering the RGB coordinates from the sensor image taken by the smartphone. Quantification analysis was performed using the ImageJ processing tool. In this case, the most sensitive color component was the red coordinate, which had a slope value of −5.9 and could be understood as a decrease in the orange coloring of the NQS-based sensor as the ammonium concentration increased in the working medium. In conclusion, reasonable calibration parameters were obtained when using smartphone-based devices, which could be employed as additional support for in situ analysis. This portability aspect confers a remarkable advantage of the proposed sensing membrane for detecting ammonium and urea. Table 7 gives the selected methodologies for these analytes, which were proposed or could be adapted for in situ analysis. From this table, it can be concluded that the proposed sensor is an environmentally friendly alternative for in situ ammonium and urea determination.

## 7. Discussion

The present work demonstrated the potential application of NQS-doped PDMS-based sensors to determine both NH_4_^+^ in water samples and urea in urine samples. The sensing device showed good precision (RSD < 8%), satisfactory stability, and promising versatility when analyzing different wastewater samples and different human urines. The colorimetric response obtained was registered by conventional diffuse reflectance measurements and smartphone-supported spectrometric measurements for quantitative analysis. In both cases, satisfactory results were obtained and showed good concordance. Moreover, semi-quantitative analysis is available by visual inspection of the sensor color, making it a user-friendly device for in situ analysis purposes. In addition to ammonia, direct urea detection was also assessed by the proposed sensing device. Urea hydrolysis was also analyzed in the presence of urease enzyme in solution and immobilized on a glass support. Both hydrolysis reactions were demonstrated to be quantitative, which increases the feasibility of the proposed sensor as a promising sensing device. Hence, the results obtained broaden the application of the NQS-based sensor to analyze amino groups present in biological matrices, such as urine, in addition to water matrices.

## Figures and Tables

**Table 1:** Calibration curves obtained for ammonia, urea, and hydrolyzed urea determination.

Analyte	Instrumentation	Linearity (y = b_0_ + b_1_x)		
		b_0_ ± s_bo_	b_1_ ± s_b1_ (L mg^−1^)	R^2^
Ammonia	Conventional	0.007 ± 0.010	0.035 ± 0.002	0.990
	Portable	0.006 ± 0.010	0.0316 ± 0.0019	0.999
	Smartphone	193 ± 4	−5.9 ± 0.7 (no units)	0.957
Urea	Conventional	0.003 ± 0.010	0.153 ± 0.008	0.995
		−0.004 ± 0.009	0.149 ± 0.005	0.998
Ammonia ^1^	Conventional	0.014 ± 0.016	0.032 ± 0.003	0.990
Ammonia ^2^	Conventional	0.002 ± 0.005	0.033 ± 0.001	0.998

^1^ Hydrolysed urea with urease in solution. ^2^ Hydrolyzed urea when urease is immobilized on a solid support.

**Table 2:** Assayed volumes from urease solution, with absorbance at 590 nm, and equivalence to ammonia concentration.

V_urease_ (µL)	Abs 590 nm	[NH_3_] (mg L^−1^)	Recovery (%)
0	0.2050	-	-
10	0.3820	5	95
20	0.4806, 0.4782	6.2, 6.1	117, 116
40	0.4984, 0.4911	6.7, 6.5	127, 123

**Table 3:** Colorimetric sensor response and found concentration from the entrance, decantation, and exit stages from water treatment plant 1 and anoxic and oxic reactors from water treatment plant 2. Sensor images from the different stages of a water treatment plant.

	**PLANT 1**	**After Dilution** **Concentration ± s**	**Real Concentration ± s**
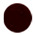	Entrance	6.15 ± 0.16	123.0 ± 3.2
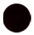	Decantation	6.99 ± 0.11	139.8 ± 2.2
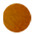	Exit	2.42 ± 0.09	4.82 ± 0.18
	**PLANT 2**	**Found Concentration (mg L^−1^) (*n* = 3)**	**Recovery (%)** **(*n* = 3)**
	Anoxic reactor	18.0 ± 0.7 ^1^	95 ± 5
		4.8 ± 0.2 ^2^	90 ± 7
	Oxic reactor	2.5 ± 0.2 ^1^	87 ± 7
		<LOD ^2^	100 ± 4

^1^ Water sample from the entrance of the treatment plant. ^2^ Water sample from the exit of the treatment plant.

**Table 4:** Absorbance values for untreated and deproteinized urine samples and found urea concentrations. Experimental scheme: direct urea → protein precipitation → sensor response.

Urine	Absorbance 590 nm		Urea Concentration (g L^−1^)
	Untreated	Deproteinized	
Sample 1	0.6615	0.3869	27.7
Sample 2	0.5654	0.2895	20.7

**Table 5:** Urea concentration found in hydrolyzed urine samples. Experimental scheme: Hydrolyzed urea → dilution → sensor response.

Urine	Urea Concentration (g L^−1^)	
	Hydrolysis in Solution	Glass Supported Hydrolysis
Sample 1	12.3	11.8
Sample 2	12.7	11.8
Average	12.5 ± 0.3	12 ± 0

**Table 6:** Ammonia concentration found in hydrolyzed urine samples by the thymol-based sensor.

Ammonia	b_1_ ± s_b1_ (L mg^−1^)	R^2^
Hydrolyzed urea	0.204 ± 0.005	0.983
Hydrolyzed urine	0.194 ± 0.006	0.999
	0.193 ± 0.007	0.999

**Table 7:** Selected methodologies for ammonium and urea determination.

AMMONIUM					
Option/Ref	Technique	Reagent(s)	Time of Analysis (s)	LOD (mg·L^−1^)	Sample
Colorimetric sensor by reagent delivering/[10,11]	UV-vis spectroscopy	PDMS, thymol or salicylate, nitroprusside	600/300	0.4/0.03	Environmental water
Ammonia selective electrode/[51]	Potentiometry	Derivatizing OPA-NAC reagents	300	0.07	Environmental water
Chemical reaction/[52]	FIA/UV-vis spectroscopy	OPA-Na_2_SO_3_ mixed reagent solution	600	0.13	Natural water
3D microfluidic paper-based device/[53]	Digital image processing/Reflectance	Nitrazine yellow (NY)/ bromothymol blue (BTB) as indicators	300	0.41/0.6	Freshwater
Ion-selective electrode (ISE)/[54]	Potentiometry	Ag/nano-Ag/polyaniline/poly (o-phenylenediamine) doped electrode	300	0.22	Tap water
Solid colorimetric sensor/this work	Diffuse reflectance spectrophotometry /digital image processing	1,2-naphthoquinone-4-sulfonate (NQS), PDMS	600	0.4	Environmental water
**UREA**					
Urea biosensor/[55]	Potentiometry	Urease, surface-modified fullerene nanomaterial	55	2.4	Urine
Urea biosensor/[56]	Amperometry	Urease, poly(3-aminopropyl-pyrrole-co-pyrrole) support, electrochemical deposition on indium-tin- oxide-coated glass	42	1.2	Human serum
Urea pH sensor/[32]	Potentiometry	Iridium oxide films, silicon-based thin-film platinum microelectrode	180	4.7	Urine
Enzymatic optical biosensor/[57]	Optical	Urease, FITC-dextran sensing probe entrapped in TMOS	600	0.15	River water, serum
Enzyme-based field effect transistors/[58]	Potentiometry	Urease, layered double hydroxide (LDH) clay matrix, glutaraldehyde cross-linker	<12	0.21	Urine, blood
Solid colorimetric sensor (this work)	Diffuse reflectance spectrophotometry/digital image processing	1,2-naphthoquinone-4-sulfonate (NQS), PDMS	600	0.4	Human urine

## Data Availability

Not applicable.
